# Functional surfaces, films, and coatings with lignin – a critical review

**DOI:** 10.1039/d2ra08179b

**Published:** 2023-04-24

**Authors:** Jost Ruwoldt, Fredrik Heen Blindheim, Gary Chinga-Carrasco

**Affiliations:** a RISE PFI AS Høgskoleringen 6B Trondheim 7491 Norway jostru.chemeng@gmail.com

## Abstract

Lignin is the most abundant polyaromatic biopolymer. Due to its rich and versatile chemistry, many applications have been proposed, which include the formulation of functional coatings and films. In addition to replacing fossil-based polymers, the lignin biopolymer can be part of new material solutions. Functionalities may be added, such as UV-blocking, oxygen scavenging, antimicrobial, and barrier properties, which draw on lignin's intrinsic and unique features. As a result, various applications have been proposed, including polymer coatings, adsorbents, paper-sizing additives, wood veneers, food packaging, biomaterials, fertilizers, corrosion inhibitors, and antifouling membranes. Today, technical lignin is produced in large volumes in the pulp and paper industry, whereas even more diverse products are prospected to be available from future biorefineries. Developing new applications for lignin is hence paramount – both from a technological and economic point of view. This review article is therefore summarizing and discussing the current research-state of functional surfaces, films, and coatings with lignin, where emphasis is put on the formulation and application of such solutions.

## Abstract 2

Lignin can be modified and used in functional surfaces, films, and coatings.

## Graphical abstract

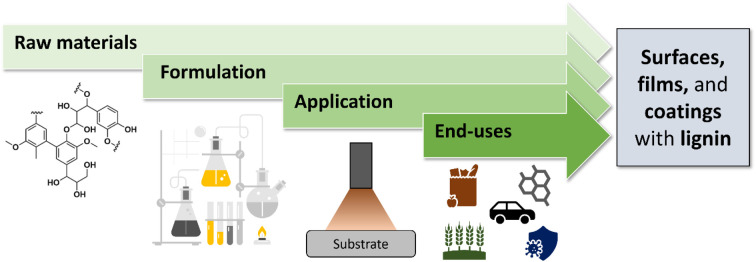

## 1. Introduction

Lignin is the second most abundant biopolymer on earth, after cellulose. Natural lignin is synthesized from the three monolignol precursors, namely *p*-hydroxyphenyl (H unit), guaiacyl (G unit), and syringyl (S unit) phenylpropanoid.^1^ Lignin from softwood consists primarily of G units, whereas hardwood lignin contains both G and S units.^2^ Moreover, lignin from annual plants, such as grass or straw, can contain all three monolignol units.

Technical lignin is the product of biomass separation processes and hence differs from natural or pristine lignin, as it is found in lignocellulose biomass.^3^ The composition and properties of technical lignin are largely determined by their botanical origin, extraction process, purification, and potential chemical modification.^4^ Presently, there are some 50–70 million tons technical lignin available from pulping or biorefinery operations. Most is burned to produce energy in biorefinery processes and only approx. 2% is sold commercially.^5^ Technical lignin isolated from pulping processes includes Kraft and soda lignin from alkali pulping, lignosulfonates from sulfite pulping, and organosolv lignin from solvent pulping.^6^ The two main types of technical lignin are lignosulfonates (approx. 1 million tons per year) and kraft lignin (<100 000 tons per year). In addition, the advent of hydrolysis and steam-explosion lignin have created new types of technical lignin.^7,8^ The use of ionic-liquids or supercritical solvents have furthermore yielded the products ionosolv lignin and aquasolv lignin, respectively, with new and interesting features.^9,10^

Lignin is polyaromatic and due to this structure, it is less hydrophilic than polysaccharidic biopolymers, *e.g.*, cellulose, hemicellulose, starch, alginate or chitosan.^11^ It is hence a promising candidate in various applications, including: (i) reduction of wettability of hydrophilic materials, (ii) addition of functionalities, such as protection from UV light, antioxidant and antimicrobial properties, and (iii) tailoring of materials and formulations, *e.g.*, for controlled substance release, adsorption, or antifouling mechanisms.^12–16^ However, chemical modification is required for most applications of lignin. Such modifications frequently make use of lignin's hydroxyl groups, for example, by grafting reactions during phosphorylation, sulfomethylation, esterification, or amination.^17^ The aromatic moieties in lignin can furthermore be targeted for, *e.g.*, replacing phenol in formaldehyde resins.^18^ At last, the carboxyl groups in lignin may also serve as reactive sites for polyesters.^19^

Interest has also been strong for the use of technical lignin in polymeric materials, *e.g.*, for thermoplastics or thermosets.^20^ Processability of lignin in thermoplastics can be done without modification, as lignin is an inherently thermoplastic material.^21,22^ Lignin's glass transition temperature can range from about 60–190 °C and may depend on many factors, including the botanical origin and pulping type, moisture content, and chemical modification.^23,24^ Lignin can also be chemically modified to improve the application of lignin as specialty chemicals or in polymeric materials.^25–27^ Additionally, the utilization of lignin as macromonomer, *i.e.*, thermoset precursor, can be done as part of polyurethanes, polyesters, epoxide resins, and phenolic resins.^11^ End-uses include the production of rigid or elastic foams, rigid and self-healing materials, adhesives, biocomposites, and coatings.^19,28–33^

One long-held belief is that lignin provides water-proofing in the wood cell wall to support water-transport.^34^ Despite yielding a contact angle below 90°, which would be required to pose as a hydrophobic material, various researchers have shown that lignin can reduce the wettability and water-uptake of wood and pulp products.^18,35–37^ Hence, both technical and chemically modified lignin have been proposed as additives for packaging materials.^38^ Reduction of wettability of fiber-based packing is a particularly interesting application, considering environmental and societal drivers regarding reduction of single-use plastics and environmental pollution. Lignin could thus form the basis of coatings or impregnation blends, provided that the lignin-coating complies with food contact requirements. One example for lignin-blends is the combination with starch during surface-sizing of paper, which can improve extensibility and reduce wetting of the starch-matrix.^35,39^ Layer-by-layer assembly with multivalent cations or polycationic polymers has also been done, which can improve the strength and hydrophobicity of cellulose.^40,41^

Other applications of lignin, its derivatives and mixtures include the use for controlled-release fertilizers, antifouling membranes, fire retardancy, dye sorption, wastewater treatment, and corrosion inhibitors.^14–16,42–44^ One publication even reported an unintentional but yet advantageous coating of coir fibers, where the lignin delayed oxidation and thermal degradation of the fibers in a polypropylene composite.^45^

Major drivers for using lignin are economical aspects by attributing value to a by-product from pulping or biorefinery operations, and sustainability by replacing fossil-based materials with biopolymers. Many applications can thus benefit from the inclusion of lignin in functional surfaces, films, and coatings. The mechanism of action and application mode can hereby differ greatly. This review therefore represents an effort to structure and summarize recent progress, where emphasis is put on both the process and final use for lignin in surfaces and coatings.

## 2. Fundamentals

### 2.1. Structure and composition of natural lignin

**Fig. 1:**
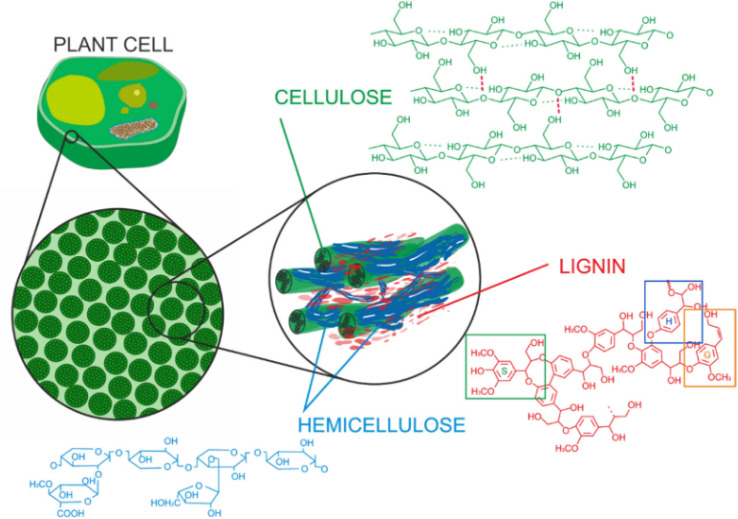
Composition of lignocellulosic biomass and the structural roles of cellulose, hemicellulose, and lignin.^46^

Lignin is part of the lignin-carbohydrate complexes (LCC) that are found in cell walls of plants and woody materials, as illustrated in Fig. 1. The cellulose fibers are tightly bound to a complex network of hemicellulose and lignin, and the three biopolymers provide strength and stability to the cell walls. In addition to providing structural integrity, lignin helps building hydrophobic surfaces which are important in transport channels for water and nutrients.^46^

**Fig. 2:**
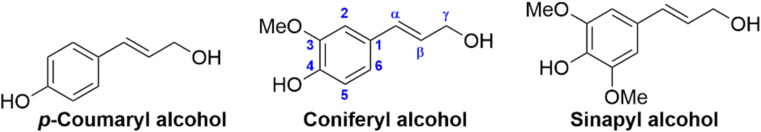
Monolignol structure and positions indicated by blue numbers and letters.

The complex lignin network consists of the three 4-hydroxyphenyl propylene units, or monolignols, formed from the parent compounds *p*-coumaryl- (*p*-hydroxyphenyl, H-unit), coniferyl- (guaiacyl, G-unit) and sinapyl alcohol (syringyl, S-unit), see Fig. 2.^47^ The monolignols differ only in the presence or absence of one or two aromatic methoxy groups *ortho* to the hydroxyl group. These are synthesized *in vivo* from the aromatic amino acid phenylalanine, formed in the shikimic acid pathway in plants.^48^ The resultant monolignols undergo a variety of radical cross-coupling reactions which results in the complex, and varied, lignin network. The ratios of the three monolignols in lignin from different sources can vary quite significantly, hardwood lignins contain G- (25–50%) and S-units (50–70%), softwood lignins contain mostly G-units (80–90%), while grass lignin contains mixtures of S- (25–50%), G- (25–50%) and H-units (10–25%).^49^

The monolignol composition (H : G : S ratio) can also vary between tissue types in the same organism, which has been illustrated in the cork oak, *Quercus suber*. Lignin from the xylem (1 : 45 : 55) and phloem (1 : 58 : 41) differ less in composition than the two compared to the phellem (cork-part, 2 : 85 : 13). These differences affect the occurrence of specific interunit linkages, where an increase in S-units lead to an increase in alkyl–aryl ether (β-O-4) bonds: 68% in cork, 71% in phloem, 77% in xylem.^50^

**Fig. 3:**
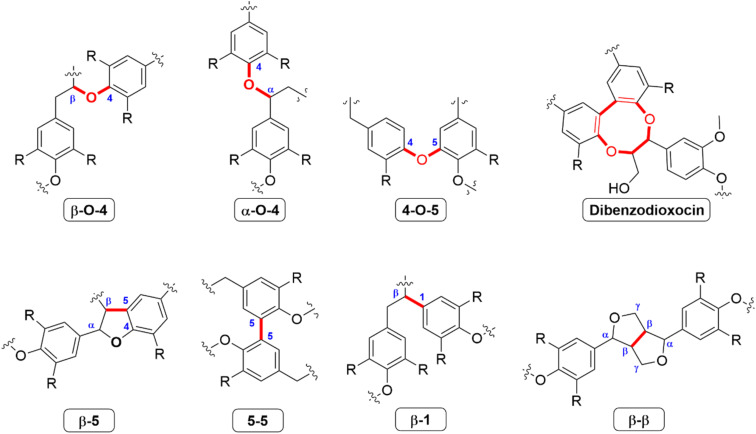
Common linkages between monolignols identified in lignins.^51,52^

The difference in abundance of the three monolignols lead to many different types of interunit linkages in lignin, specifically between angiosperm (hardwood and grass) and gymnosperm (softwood) lignin.^51^ The most common interunit linkage is the β-O-4 alkyl–aryl ether bond (Fig. 3), which occurs between 45–50% or 60–62% of phenyl propylene unit (C_9_ units) in softwoods and hardwoods, respectively. As this is the most common linkage, many delignification processes target this specific linkage. For softwoods, the 5–5 linkage is also important, and has an abundance of 18–25% per 100 C_9_ units, while this linkage occurs only around 3–9% in hardwoods.^52^

**Fig. 4:**
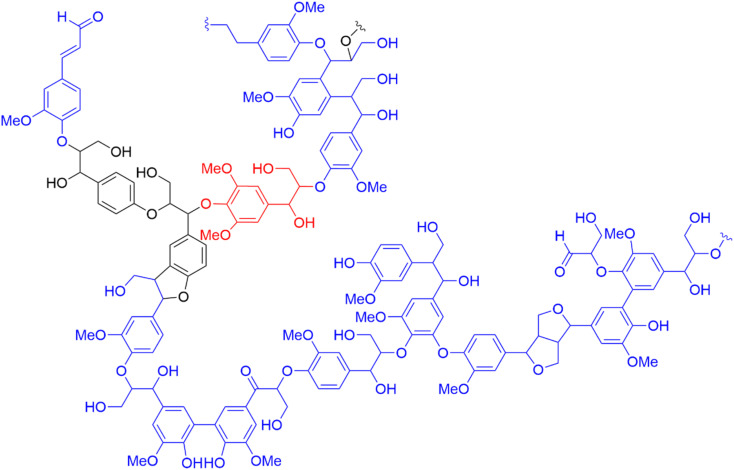
Adaptation of Adler's representation of softwood (spruce) lignin with color-coded monolignols: *p*-hydroxyphenyl (H-unit) in black, guaiacyl (G-unit) in blue and syringyl (S-unit) in red.^55^

In the process of isolating technical lignins, both the labile aryl-alkyl and β-O-4 bonds are most prone to cleavage.^53^ This results in technical lignins having more condensed and variable structures than native lignin, and a wide variety in molecular weight (*M*_w_). Mass average values (*M*_w_) of 1000–15 000 g mol^−1^ for soda lignin, 1500–25 000 g mol^−1^ for Kraft lignin, and 1000–150 000 g mol^−1^ for lignosulfonates have been reported, depending on botanical origin and process conditions.^54^ Native lignin is a virtually infinite macromer that is both randomly- and poly-branched.^55^ The bonds between the lignin and surrounding hemicellulose and cellulose found in LCC have recently been reviewed.^56^ All softwood lignin, and 47–66% of hardwood lignin, is reportedly bound covalently to carbohydrates, and mainly to hemicellulose. The most common types of linkages found in LCCs are benzyl ether-, benzyl ester-, ferulate ester-, phenyl glycosidic- and diferulate ester bonds.^57^ Note that due to the high degree of variability in inter-unit and LCC linkages, Fig. 4 should only be taken as an illustrative example. The lignin macromolecule is polydisperse and may exhibit various linkages and functional groups.^4^ In other words, lignin should be considered as statistical entities rather than distinct polymers.

### 2.2. Isolation of technical lignin

**Fig. 5:**
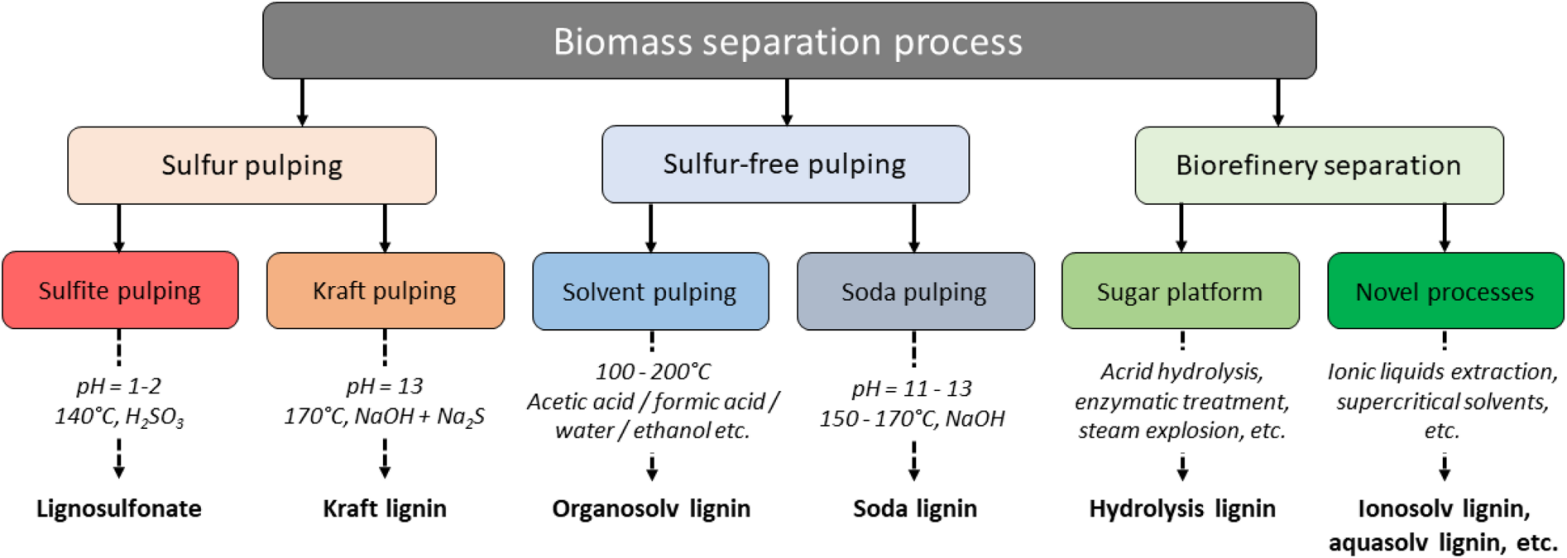
Lignin extraction processes and their products.^3^

Lignocellulosic biomass consists of cellulose (30–50%), hemicellulose (20–35%) and lignin (15–30%), where the lignin acts as a “glue” within the LCCs.^58,59^ The actual lignin content of the biomass is highly influenced by its botanical origin *e.g.*, 28–32% is found in pine and eucalyptus wood, while switchgrass contains only 17–18% lignin,^60^ and less than 15% is typically found in annual plants.^61^ The first step in lignin valorization is biomass fractionation, where the cellulose, hemicellulose, and lignin are separated from each other. Several techniques have been developed, which can be grouped into sulfur and sulfur-free pulping from the paper and pulp industry, and biorefinery processes that aim to produce of materials, chemicals, and energy from biomass.^59^ The latter may specifically be designed to isolate lignin of high purity and reactivity, whereas pulping originally produced lignin as a by- or waste-product. An overview is given in Fig. 5.

The three industrial extraction methods for lignin are kraft, sulfite and soda pulping. In addition, organosolv pulping has been developed to extract lignin and separate the pulp fibers. Commercialization of this process has not yet been done, but interest has risen recently in this technology, as organosolv pulping produces a technical lignin of high purity and reactivity. Several other methods also exist, but these are mainly used in lab-scale and are referred to as biorefinery concepts, or “pretreatments”.^62^ In the Kraft pulping process, the lignocellulosic biomass is mixed with a highly alkaline cooking liquid containing sodium hydroxide (NaOH) and sodium sulfite (Na_2_S), at elevated temperatures of 150–180 °C. From the resulting black liquor, kraft lignin can be precipitated out by lowering the pH to around 5–7.5.^46^ In the LignoBoost process, this precipitation is done by adding first CO_2_ and then sulfuric acid. Kraft lignin has a sulfur content of 1–3%, is highly condensed, contains low amounts of β-O-4 linkages, and is frequently burned for energy and chemical recovery at the mills.^63–65^ In the Kraft process, lignin is fragmented through α-aryl ether or β-aryl ether bonds, which results in increased phenolic OH content in the resultant lignin.^66^ The sulfite process is another specialized pulping technique, which utilizes a cooking liquor containing sodium, calcium, magnesium or ammonium sulfite and bisulfite salts.^65^ Treatments are typically conducted at 120–180 °C under high pressures, which gives lignosulfonates that contain 2.1–9.4% sulfur, mostly in the benzylic position.^67^ Lignosulfonates are cleaved mainly through sulfonation at the α-carbon, which leads to cleavage of aryl-ether bonds and subsequent crosslinking.^68^ Both Kraft and sulfite black liquor typically contain significant amounts of carbohydrate and inorganic impurities.^64^ The soda-anthraquinone process is mostly applied in the paper industry on non-woody materials like sugarcane bagasse or straw.^64^ The material is treated with an NaOH solution (13–16 wt%) at high pressures and temperatures of 140–170 °C, where anthraquinone is added to stabilize hydrocelluloses.^64,69^ The resulting soda lignin is sulfur-free and contains little hemicellulose or oxidized moieties. Organosolv lignin is produced in an extraction process using organic solvents and results in separation of dissolved and depolymerized hemicellulose, cellulose as residual solids, and lignin that can be precipitated from the cooking liquor.^65^ Various solvent combinations are possible, such as ethanol/water (Alcell process) or methanol followed by methanol and NaOH and antraquinone (Organocell process), which will affect structure of the resultant materials.^51^ Common for all organosolv lignins is that their structures are closer to that of natural lignins, in particular compared to Kraft lignin or lignosulfonates. They are additionally sulfur-free and tend to contain less than 1% carbohydrates.^65^

Several other methods of biomass processing have been developed that are targeted at lignin extraction, rather than producing cellulose fibers, where lignin is as a byproduct.^64^ Milled wood lignin (MWL) can be produced to closely emulate native lignin, but at the expense of process yields.^70^ This method is considered gentle but time consuming, often requiring weeks of processing, making it viable only in a laboratory setting.^58^ Other techniques that aim to produce native lignin analogues include cellulolytic enzymatic lignin (CEL) and enzymatic mild acidolysis lignin (EMAL). The CEL procedure was developed as an improvement of the MWL process, where higher yields were obtained without increasing milling duration.^46^ By adding an additional acidolysis step, Guerra *et al.* were able to again improve on the yield, while still producing lignin that closely resembled the native structure.^70^ The physicochemical pretreatments aim to reduce lignin particle size through mechanical force, extrusion, or other. These techniques include steam explosion, CO_2_ explosion, ammonia fiber expansion (AFEX) and liquid hot water (LHW) pretreatments.^46^ Ionic liquids have also been successfully used for lignin isolation. Five cations with good solubilizing abilities were identified: the imidazolium, pyridinium, ammonium and phosphonium cations, while the two large and non-coordinating anions [BF_4_]^−^ and [PF_6_]^−^ were found to disrupt dissolution of the lignin.^46^ The chosen extractive method will not only affect the characteristics of the resulting lignin, but also the amount that is extracted. Several methods have been developed for the delignification of sugarcane bagasse, *e.g.*, milling, alkaline or ionic liquid extraction, where yields of 17–32% were obtained depending on the method of choice.^71^

### 2.3. Chemical modification

**Fig. 6:**
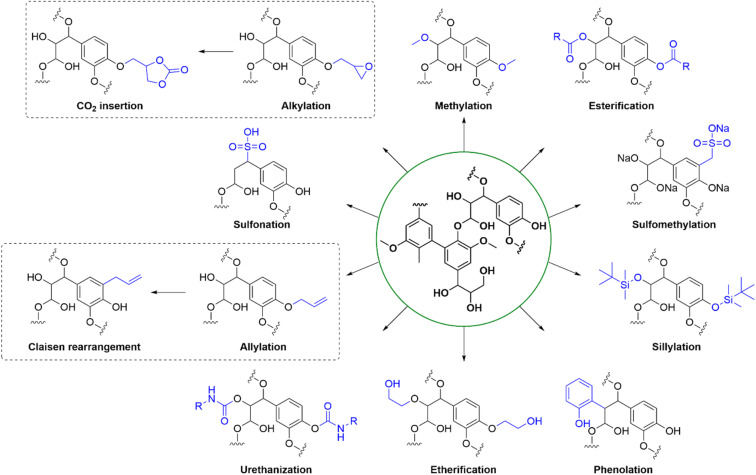
Examples of chemical modifications of technical lignin.

Chemical modification of technical lignins is well explored and include a huge variety of techniques (see Fig. 6 for illustrative examples). Technical lignins have been modified by a myriad of techniques, such as esterification, phenolation and etherification.^6^ Urethanization with isocyanates has been explored towards polyurethan production,^72^ and allylation of phenolic OH groups enabled Claisen rearrangement into the *ortho*-allyl regioisomer which is of interest for its thermoplastic properties.^73^ The solubility and charge density of technical lignins can be affected by sulfomethylation or sulfonation,^17,74^ and methylation of the phenolic OH groups have led to lignin with an increased resistance to self-polymerization^17^ The thermal stability of lignins has also been improved by silylating the hydroxyl groups with TBDMS-Cl, and the resulting material could be incorporated into low-density polyethylene (LDPE) blends forming a hydrophobic polymer matrix.^75^ Lignin is a versatile scaffold for different modifications depending on the desired application. For the production of epoxy resins, epoxidation with epichlorohydrin is a common technique. This approach has also been combined with CO_2_ fixation resulting in cyclic carbonates being incorporated in the lignin.^76^

### 2.4. Analysis techniques

Techniques to assess lignins and lignocellulosic biomass have long been a topic of great interest, both for quantitative and qualitative characterization. Such techniques are also critical to probe and assess chemical modifications. A summary of common methods is given in Table 1.

**Table 1:** Characterization techniques to assess lignins quantitatively and qualitatively

Method	Description	Limitations	Ref.
FTIR spectroscopy	Popular technique to combine with chemometric methods such as principal component regression (PCR) or partial least squares (PLS) regression. Have been used for successfully determining lignin content in biomass samples	Calibration required with samples of known concentrations. Large dataset (training and test sets) needed for reliable quantification. Training samples and prediction samples cannot differ greatly. Analyses are sensitive to sample preparation techniques	76 and 77
^1^H/^13^C 2D NMR	Extremely detailed information about inter-unit linkages can be obtained. Has allowed for the assignment and quantification of over 80% of linkages in lignin oil from reductive catalytic fractionation of pine wood	NMR experiments are expensive, instruments found at specialized institutions and universities. Both experiments and data processing can be highly time-consuming	78 and 79
^31^P NMR	Differentiation of the phenolic OH content of the three monolignols is possible from experiments after derivatization of the OH groups	Full derivatization of OH-groups is essential for proper quantification. Inverse gated decoupling pulse sequence needed for quantification: reduced sensitivity and increases relaxation time of analysis	78
UV-vis spectroscopy	Cruder determination of phenolic OH content is possible by comparing the differences in absorption at specific maxima between neutral and alkaline solutions	Less accurate than ^31^P NMR. Affected by incomplete ionization of functional groups. Presence of other ionizable groups can affect results	78 and 80
Simultaneous conductometric and acid–base titrations	A fast and cheap alternative to wet-chemical methods for determining both phenolic OH group and carboxylic acid contents	Heterogeneity of COOH- and OH-groups distorts inflection point. Limited to quantification of COOH- and OH-groups (and possibly other ionizable groups)	81 and 82
Size exclusion chromatography (SEC)	Popular technique for obtaining weight and number average molecular weights, *M*_w_ and *M*_n_, and for further calculating the polydispersity index (PI) of samples	Time consuming calibration required. Samples must be within linear range. Acetylation is often used for increased solubility prior to analysis	78 and 83
Gas chromatography – pyrolysis (GC-Py)	Analysis of biomass composition, quantification of volatiles, bio-oil and biochar. Can be coupled with TGA and FTIR.	Variations in inherent metal contents greatly affects the pyrolysis reaction of the biomass	84 and 85
Coherent anti-Stokes Raman scattering (CARS) microscopy	Label-free method with high sensitivity and chemical selectivity for imaging of lignin in *e.g.* plant cell walls	Interference from other aromatic compounds (phenylalanine, tyrosine) can distort image. Not suited for imaging all tissue types	58 and 86
Time-of-flight secondary ion mass spectrometry (ToF-SIMS)	Visualization of monolignol distribution on plant sample cross-sections	Prone to artefact-generation arising from sample preparation. Fragmentation of lignin during imaging due to high-energy ion bombardment	87

Different techniques are often combined to provide a better overall picture. For example, chemical modification of lignin may be probed in terms of molecular weight, *i.e.*, by using size-exclusion chromatography, and abundance of functional groups, as determined by FTIR or 2D NMR analysis. The technique of choice can depend on factors such as the target groups of interest, but also on availability and cost. The polydisperse nature of technical lignin can sometimes make accurate measurements difficult. This is manifested, for example, in the incomplete ionization of phenolic moieties during titration or UV spectrophotometry, as the configuration and side chains of phenolic moieties induce varying degrees of resonance stabilization.

## 3. Formulations and applications of lignin-based surfaces and coatings

The coatings and surface modifications in this review most often fulfill one of two purposes. Firstly, they may seek to protect the underlying substrate, *e.g.*, from mechanical wear, chemical attack (corrosion), or UV radiation. Secondly, they add functionality such as antioxidant, controlled substance release, or antimicrobial properties. Reduced wetting and hydrophobization are frequently mentioned for lignin,^34,77,78^ which would normally fall into the second category, unless the purpose is to protect the underlying substrate from degradation by water. The different applications will be discussed more in detail in this chapter.

**Fig. 7:**
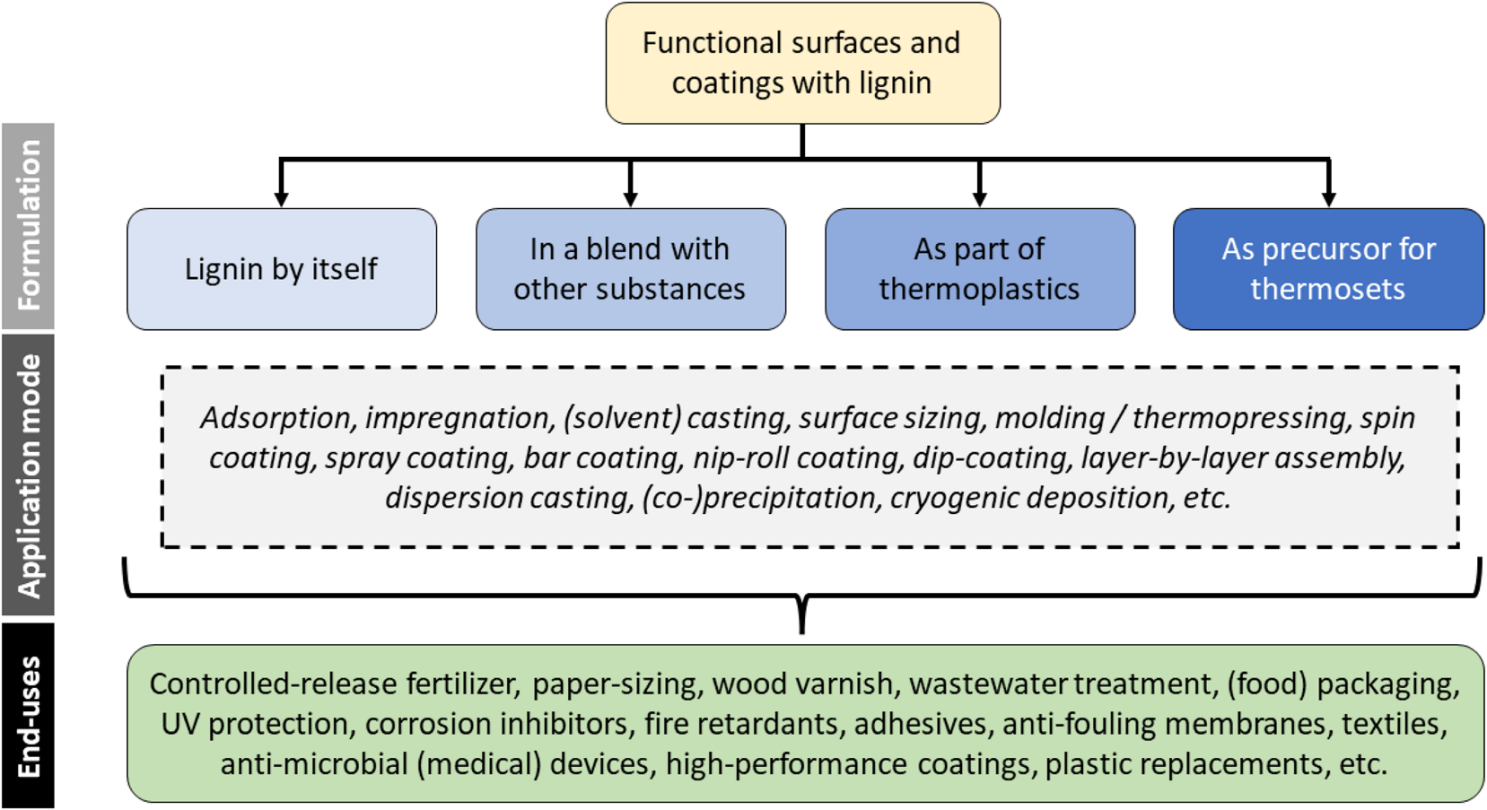
Overview of different application modes for producing functional surfaces and coatings with lignin.

The end-use usually determines the manner, in which mixtures and coatings must be formulated. In principle, four different approaches can be distinguished, which are (1) application of neat lignin, (2) blends of lignin with other active or inert materials, (3) the blending of lignin in thermoplastic materials, and (4) the use of lignin as a precursor for synthesizing thermoset polymers. An overview of the different approaches for formulation and application is given in Fig. 7. These will be discussed in more detail further on.

While surface layer or coating are usually applied onto another material, there are also implementations that include lignin as part of the overall base-matrix. Examples for the latter include lignin-derived biocarbon particles for CO_2_ capture or wastewater treatment, polyurethane foams, and lignin as an internal sizing agent in pulp products.^36,43,79,80^ The predominant way of using lignin in functional surfaces is by blending with other substances. Such formulations often include agents, which are established for a particular application, *e.g.*, starch for paper sizing or clay for controlled-release urea fertilizers.^81,82^ Formulations in polymer synthesis usually draw on specific functional groups that are found in lignin, for example, the hydroxyl groups as polyol replacement in polyurethane or the aromatic moieties as phenol replacement in phenol-formaldehyde resins.^18,33^

### 3.1. Surfaces and coatings with neat lignin

Applying technical lignin by itself is a simple approach, as no co-agents are required. While some degree of adhesion to the substrate is often given, pressure and heat may be applied in addition. Publications pertaining to this topic can be grouped into two categories, *i.e.*, fundamental research studying the formation and properties of lignin-based films and coatings, as well as applied research, which is usually focused on a specific end-use.

#### 3.1.1. Fundamental research

**Fig. 8:**
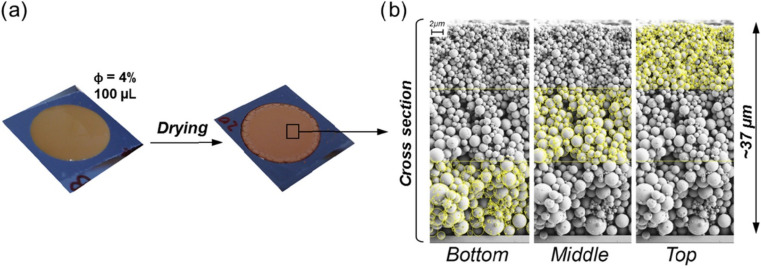
Coatings comprising lignin particles produced by evaporation-induced self-assembly (a) and vertical cross-section of the obtained layers (b).^85^

A fundamental study was performed by Borrega *et al.*, who prepared thin spin-coated films from six different lignin samples in aqueous ammonium media.^83^ The films exhibited hydrophilicity with contact angles ranging from 40–60°. Despite widely diverse compositions, the solubility in water was found to be the parameter governing the properties of the thin films. Similar results were obtained by Notley and Norgren, who found that lignin coatings prepared from diiomethane or formamide yielded even lower contact angles at about 20–30°.^34^ The approach was further refined by Souza *et al.*, who treated the spin-coated lignin films *via* UV radiation or SF6 plasma treatment in addition.^84^ While the UV treatment reduced the contact angle from about 90° to 40°, the plasma treatment produced superhydrophobic surfaces with contact angles exceeding 160°. The latter was also shown to induce major surface restructuring with a strong incorporation of CF_*x*_ and CH_*x*_ groups, which would account for the large increase in contact angle. Coatings with lignin-based nanoparticles can also be made by evaporation-induced self-assembly, whose properties and morphology are strongly governed by the drying conditions and evaporation rate.^85^ An example of the obtained morphologies is shown in Fig. 8. Based on these reports, research is generally concurring on the fact that lignin by itself is not a hydrophobic substance. Harsh treatments, chemical modifications, or fine-tuning of surface morphology are necessary to invoke hydrophobicity.

Spin-coated films of milled-wood lignin have furthermore been investigated for enzyme adsorption.^86^ Similarly, the adsorption of proteins on colloidal lignin has been studied by Leskinen *et al.*, who produced protein coronas on the lignin particles *via* self-assembly.^87^ The authors further showed that this deposition was governed by the amino acid composition of the protein, as well as environmental parameters such as the pH and ionic strength. The use of lignin for protein-adsorption is an interesting implementation, as it can provide different surface chemistries than its lignocellulosic counterparts. Still, the compatibility with *in vivo* environments is questionable, as biodegradation is not given here.

#### 3.1.2. Applied research

An example for applied research would be paper and pulp products, which can be rendered less hydrophilic by surface-sizing. Application of the lignin can be done *via* an aqueous dispersion or alternatively by impregnation after dissolution in a solvent.^35,88^ A similar approach was used to treat beech wood with lignin nanoparticle *via* dip-coating, which improved the weathering resistance of the wood.^89^ Such dip-coating may preserve breathability of the substrate due to the porous structure. In this context, the patent application WO2015054736A1 should be mentioned, which discloses a waterproof coating on a range of substrates including paper.^90^ In this invention, the lignin is coated onto the substrate after at least partial dissolution, followed by heat or acid treatment. However, as discussed above, the lignin by itself is not a hydrophobic material. While lignin-nanoparticles may alter the surface morphology of pulp products, an improvement in long-term water-resistance may be mostly determined by affecting mass-transfer kinetics.

Deposition of lignosulfonates on nylon has been demonstrated, which improved the ultraviolet protection ability of the fabric.^91^ This deposition took place from aqueous solution and under heating, reportedly yielding a chemical bonding of lignin's OH groups to the NH groups of nylon 6. Such bonding would indeed be necessary, as the lignosulfonate would otherwise be easily washed away.

Zheng *et al.* coated microfibrillated cellulose with Kraft lignin and sulfonate Kraft lignin, which promoted fire retardancy of the material.^42^ At last, iron-phosphated steel was rendered more resistant to corrosion after spray coating with lignin, which was first dissolved in DMSO and other commercial lignin-solvents.^92^ While proven in the lab, these two applications must be considered with care, as unmodified lignin is a brittle material, which can limit the long-term durability of such products.

### 3.2. The use of chemically modified lignin

Chemical modification of lignin is frequently done to improve or enable the processability in blends with materials. In addition, chemical modification may add or alter functionalities as required in specific applications.

#### 3.2.1. Lignin-ester derivatives

**Fig. 9:**
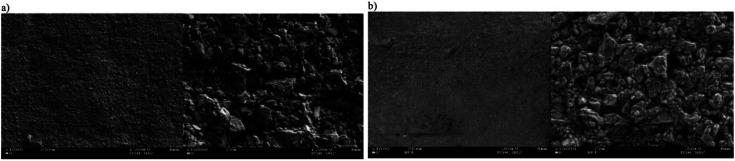
SEM images of (a) uncoated paperboard and (b) paperboard coated with lignin-fatty acid ester. This figure has been adapted/reproduced from ref. 78 with permission from Elsevier B.V., copyright 2013.

Esterification of lignin with fatty acids has been investigated by several authors. This approach bears potential, as it combined two bio-derived (macro-)molecules. The lignin contributes a backbone for grafting and may improve dispersibility and adhesion of the fatty acids on lipophobic surfaces. The fatty acids can in turn render the lignin more hydrophobic, improving the water barrier, *e.g.*, on paper substrates. To improve the reaction yield, reactive intermediates are frequently used. Several publications have studied the use of lignin esterified with fatty acid-chlorides as hydrophobization agents for paper and pulp products.^78,93^ The coating affected both the surface chemistry and morphology, as illustrated in Fig. 9. The result is usually a decrease in water-vapor transmission rate (WVTR), oxygen transmission rate (OTR), and an increase in aqueous contact angle. Oxypropylation with propylene carbonate has been used as an alternative esterification approach, which yielded a similar hydrophobization and barrier effect on recycled paper.^94^ A downside of oxypropylation is the use of toxic reactants, *i.e.*, propylene oxide, and the requirement for high pressure during the reaction. While fatty acid chlorides do not need high pressures, these chemicals are highly corrosive and require the absence of water. All mentioned aspects can stand in the way of commercial implementation.

Hua *et al.* reacted softwood Kraft lignin with ethylene carbonate to convert phenolic hydroxyl units to aliphatic ones,^95^ as these are considered more reactive. The samples were further esterified with oleic acid and spin- or spray-coated onto glass, wood, and Kraft pulp sheets. The authors showed that hydrophobic surfaces with contact angles ranging from 95–147° were possible. The pulp boards furthermore showed a more uniform surface after the coating. Esterification with lauroyl chloride was also used by Gordobil *et al.*, who studied their application as wood veneer by press-molding and dip-coating.^96^

While the feasibility to treat wood and wood-based products was demonstrated on a technological level, the comparison to established treatment agents is frequently lacking. For example, linseed oil is an established wood-treatment agent, which undergoes self-polymerization in the presence of air. Paper-sizing agents can be based on compounds that are similar in function to fatty acids, such as resin acids or alkenyl succinic anhydride. Considering these examples, the questions arises whether modifying lignin bears an advantage over using established coatings or sizing agents. In the light of this discussion, the acid-catalyzed transesterification of lignin with linseed oil should be mentioned.^77^ According to the authors, a suberin-like lignin-derivative was produced, which introduced hydrophobicity on mechanical pulp sheets, while being more compatible with the fibers than linseed oil alone. The proposed process is simple in setup and reactants, which facilitates ease of implementation. In addition, the lignin is prescribed a key function, *i.e.*, acting as a compatibilizer between the fibers and the triglycerides.

At last, controlled-release fertilizers with lignin-fatty acid graft polymers have been proposed. Wei *et al.* crosslinked sodium lignosulfonate with epichlorohydrin, followed by esterification with lauroyl chloride.^97^ Sadehi *et al.* reacted lignosulfonate with oxalic acid, proprionic acid, adipic acid, oleic acid, and stearic acid.^98^ The modified lignin was further used to spray-coat urea granules. Both implementations showed enhanced hydrophobicity and the ability to coat urea for slower release of nitrogen. Still, it would be important to compare such approaches with established coating or blends of lignin and natural waxes or triglycerides, which do not require an elaborated synthesis.

#### 3.2.2. Enzymatic modification

Enzymatic modification of lignin has the advantage of comparably mild reactions conditions, which can have a positive impact on process economics. On the downside, enzymes are comparably expensive and imposes higher technological demands. In addition, the variety of lignin-compatible enzymes is somewhat limited. Enzymatic treatment can induce a number of changes to lignin, such as oxidation, depolymerization, polymerization, and grafting with other components.^99^ For example, Mayr *et al.* coupled lignosulfonates with 4-[4-(trifluoromethyl)phenoxy]phenol using laccase enzymes.^100^ After successful coupling, the lignosulfonate films exhibited reduced swelling and an increase in aqueous contact angle. Fernandez-Costas *et al.* performed laccase-mediated grafting of Kraft lignin on wood as a preservative treatment.^101^ While the reaction itself was deemed a success, the desired antifungal effect was only obtained after inclusion of additional treatment agents, such as copper. It is hence questionable if enzymatically coupled lignin poses as a competitive wood-treatment agent, as the lignin could also be used in wood-varnish formulations with a higher technological maturity.

#### 3.2.3. Other approaches

A variety of other modifications has been proposed to develop coatings from lignin. For example, Dastpak *et al.* reacted lignin with triethyl phosphate to spray-coat iron-phosphated steel for corrosion protection.^44^ Coating of aminosilica gel with oxidated Kraft lignin was performed by electrostatic deposition, which improved the adsorption capacity for dyes from wastewater.^102^ Wang *et al.* phenolated lignosulfonate, followed by Mannich reaction with ethylene diamine and formaldehyde to produce slow-release nitrogen fertilizers.^103^ The final product exhibited elevated contact angles, however, an increased surface roughness likely also contributed to this effect, as the phenolated and aminated lignin exhibited nanoparticle structures. A different approach was taken by Behin and Sadeghi, who acetylated lignin with acetic acid to coat urea particles in a rotary drum coater.^104^ The use of lignin in slow-release fertilizers can be useful, as lignin can have a soil-conditioning effect. However, biodegradability also must be considered, which can be negatively affected by chemical modification.

Self-healing elastomers were synthesized by Cui *et al.*, who grafted lignin with poly(ethylene glycol) (PEG) terminated with epoxy groups.^31^ The authors concluded that a new material was developed with potential application for adhesives, but the ultimate stress was comparably low at 10–12 MPa. The material was named as a self-healing elastomer; however, the appearance and rheological properties suggest a thixotropic gel instead.

### 3.3. Blends of lignin with other substances

In the context of this review, the largest number of publications was found for lignin-blends with other substances. The advantage of this approach lies in the ease of implementation, flexibility for later adjustments, and potential synergies with other co-agents. The lignin and other additives may be mixed right before or during surface modification, hence not requiring lengthy preparations such as the synthesis of chemically modified lignin or a pre-polymer. To facilitate better overview, this section was subdivided into several sub-section, which were distinguished by the application area or formulation-approach.

#### 3.3.1. Cellulose fibers and other wood-based products

The use of lignin in combination with cellulose fibers, fibrils, or derivatives has received considerable attention, as this can yield all-biobased materials and coatings. For example, eucalyptus Kraft lignin and cellulose acetate were combined in solution and cast onto beech-wood, which produced a protective coating similar to bark.^37^ However, the authors did not determine the mechanical properties of the product, which would be important to address, as the potential brittleness could impart practical use. On the other hand, the biodegradation of lignin is indeed more challenging than that of cellulose and hemicellulose,^105^ which may hence contribute to an improved resistance against certain fungi and bacteria. In addition, the lignin-based veneer may add functionalities such as water-repellence, UV-protection, and improved abrasion resistance,^106^ but still a comparison with established treatment agents is lacking.

**Fig. 10:**
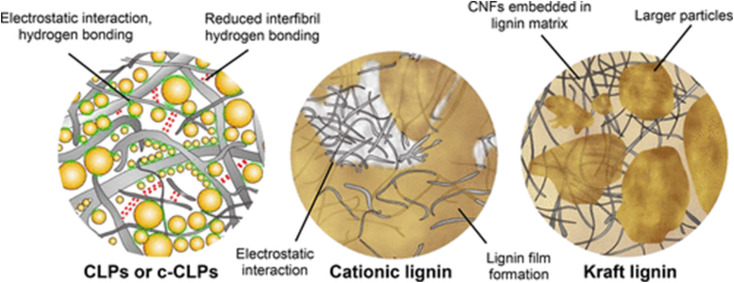
Schematic illustration of proposed interaction between CNF and different lignin morphologies.^107^

Cellulose nanofibrils (CNF) and (cationic) colloidal lignin particles was cast into films by Farooq *et al.*, yielding improved mechanical strength as compared to the CNF alone.^107^ A schematic of the proposed interactions is given in Fig. 10. The authors concluded that the lignin particles acted as lubricating and stress transferring agents, which additionally improved the barrier properties. The discussed effects could also be induced by the lignin acting as a binder, hence filling gaps and providing an overall tighter network.^36,88^ Riviere *et al.* combined lignin-nanoparticles and cationic lignin with CNF, however, the oxygen barrier and mechanical strength were lower than the CNF without added lignin.^108^ This effect was likely due to a disruption of the binding between CNF networks. The polyphenolic backbone of lignin generally provides less opportunities for hydrogen bonding than compared to the cellulose macromolecule. The authors work on solvent extraction of lignin from hydrolysis residues is noteworthy, however, and the work showed promising potential for antioxidant use.

LCC were combined with hydroxyethyl cellulose, producing free-standing composite films.^109^ In this study, the addition of LCC enhanced the oxygen barrier properties and could also improve the mechanical stability and rigidity. A better effect of LCC was noted than combining lignosulfonates with hydroxyethyl cellulose alone. Synergies could hence arise from carbohydrates that are covalently bond onto the lignin.

An interesting approach was taken by Hambardzumyan *et al.*, who Fenton's reagent to partially graft organosolv lignin onto cellulose nanocrystals.^110^ The product was cast into thin films, which showed nanostructured morphologies with increased water resistance and the ability to form self-supported hydrogel-films. In another publication, Hambardzumyan *et al.* simply mixed the cellulose nanocrystals with lignin in solution, after which films were cast onto quartz slides and dried by evaporation.^111^ The authors found that optically transparent films with UV-blocking ability could be produced. It was concluded that increasing the CNF concentration allowed for better dispersion of the lignin macromolecules, dislocating the π–π aromatic aggregates and hence yielding a higher extinction coefficient.

An elaborate work on lignin-starch composite films was conducted by Baumberger.^39^ The films were produced *via* one of two methods: (1) powder blending of thermoplastic starch and lignin, followed by heat pressing and rapid cooling, and (2) dissolution in water or dimethyl sulfoxide followed by solvent-casting and solvent evaporation. The author concluded that the lignin acted either as filler or as extender of the starch matrix, where the compatibility was favored by medium relative humidity, high amylopectin/amylose ratios, and low molecular weight lignin. Lignosulfonates formed good blends and imparted a higher extensibility onto the starch films, likely due to beneficial interactions between sulfonic and hydroxyl groups. Non-sulfonated lignin, on the other hand, improved water-resistance to a greater extent.

Three recent studies have found that incorporating lignin into a molded pulp materials can reduce the wettability of the material, as witnessed by an increase in contact angle or a decrease in water-uptake.^8,36,88^ The advantage of such implementation is that high temperature and pressure will promote densification, as the lignin can flow into cavities. High densities of up to 1200 kg m^−3^ were reported, where the uptake of water is hindered not only by limiting mass-transport, but also by confining the swelling of cellulose fibers.^88^

Various researchers have included lignin in the formulation of paper-sizing agents. In one implementation, Javed *et al.* blended Kraft lignin with starch, glycerol, and ammonium zirconium carbonate to produce self-supporting films and paperboard coatings.^112^ The mechanical film stability was better when using ammonium zirconium carbonate as a cross-linking agent, in addition to reducing the water-transmission rate. Both the lignin and the ammonium zirconium carbonate also reduced leaching of starch when in contact with water. In a second publication, the author further developed the formulation's use in pilot trials.^81^ Johansson *et al.* coated paperboard, aluminium foil, and glass with mixtures of latex, starch, clay, glycerol, laccase enzyme, and technical lignin.^113^ The authors found that the oxygen scavenging activity was greatest for lignosulfonates, as compared to organosolv, alkali or hydrolysis lignin. This effect was explained by a greater ability of the laccase to introduce cross-linking on the lignosulfonate macromolecules. In another publication, Johansson *et al.* also combined lignosulfonates with styrene-butadiene latex, starch, clay, glycerol, and laccase enzyme.^114^ The results showed that both active enzyme and high relative humidity were necessary for good oxygen scavenging activity. Laccase-catalyzed oxidation of lignosulfonates furthermore resulted in increased stiffness and water-resistance of the starch-based films. Winestrand *et al.* prepared paperboard coatings using a mixture of latex, clay, lignosulfonates, starch, and laccase enzyme.^115^ The films showed improved contact angle with active enzyme and oxygen-scavenging activity for food-packaging applications. While the results for paper-sizing with addition of lignin show promising potential, food packaging applications may impose additional requirements. For example, stability of the coatings may not be given in environments that contain both moisture and lipids. In addition, to the best of our knowledge, no study addressed the migration of sizing-agents into food. Still, the utilization of lignin as oxygen scavenger is promising, as this utilizes one of lignins inherent properties, which are found in few other biopolymers.

As an alternative to technical lignin, Dong *et al.* applied alkaline peroxide mechanical pulping effluent in paper-sizing, which comprised 20.1 wt% lignin and 16.5 wt% extractives based on dry matter weight.^116^ Blended with starch, the effluent improved the tensile index and reduce the Cobb value of paper, while providing contact angles of 120° and higher. Such implementation can, however, also aggravate certain properties of the paper, as aging and yellowing may be promoted by the presence of acids and chromophores.

Layer-by-layer self-assembly was used by Peng *et al.* to produce superhydrophobic paper coated with alkylated lignosulfonate and poly(allylamine hydrochloride).^40^ Alternatively, Lit *et al.* deposited such layers on cellulose fibers by combining lignosulfonates with the divalent copper cation instead of a polycation.^41^ A similar effect on surface morphology was noted, while contact angles within the hydrophobic regime could be achieved. The utilization of lignosulfonate-polycation assemblies for cellulose hydrophobization is somewhat counterintuitive, since polyelectrolyte complexes tend to be hydrophilic and can swell in water. The long-term stability of such coatings in water is hence questionable, still for short contact times the modulation of surface roughness and chemistry can be beneficial.

Solvent casting was employed by Wu *et al.*, using ionic liquids to dissolve cellulose, starch, and lignin.^117^ The biopolymers were coagulated by addition of the non-solvent water, further being processed into flexible amorphous films. The process appears similar to the production of cellulose regenerates. Utilizing other biopolymers than cellulose, *i.e.*, lignin, hemicellulose, and starch, is an interesting approach for fine-tuning the desired film properties.

**Fig. 11:**
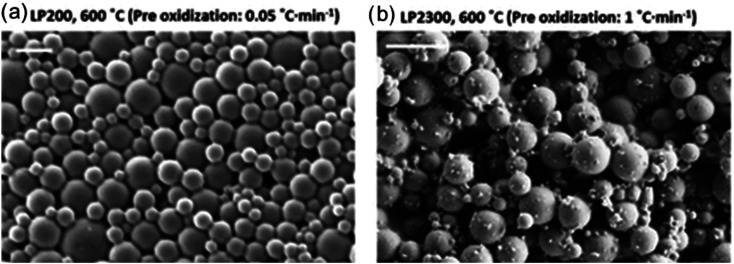
Lignin-based porous particles obtained by oxidation and carbonization for carbon capture. SEM images of lignin particles carbonized at low (a) and high (b) pre-oxidation rate, yielding two distinct morphologies. This figure has been adapted/reproduced from ref. 79 with permission from Elsevier B.V., copyright 2017.

Zhao *et al.* used evaporation induced self-assembly of lignin nanoparticles and CNF, which were subsequently oxidized at 250 °C and then carbonized at 600–900 °C.^79^ These nano- and micro-sized particles could be used for CO_2_ adsorption, where synergistic effects between the CNF and lignin nanoparticles were noted. An illustration of the particles is shown in Fig. 11.

Agrochemical formulations with lignin-based coatings predominantly involve fertilizer formulations, *i.e.*, for controlled release of nutrients. The lignin can be part of a coating, which then acts as a mass-transfer barrier that delays the dissolution of nutrients.^82,118,119^ The focus is usually on urea as nitrogen fertilizer or calcium phosphate as superphosphate fertilizers.^82,118–120^ An advantage of using lignin, apart from being biodegradable and water-insoluble, is the potential function as soil amendment.^121^

Two approaches can generally be distinguished, based on either the use of neat or chemically modified lignin. Properties such as water-permeability and nitrogen or phosphor release can be positively affected; however, chemical modification may impair biodegradation. With that said, the work of Fertahi *et al.* should be noted, who coated triple superphosphate fertilizers with mixtures of carrageenan, PEG, and lignin.^118^ The latter had been obtained from alkali pulping of olive pomace. The three mentioned coating-materials are in principle all biodegradable. Blending lignin with carrageenan or PEG improved the mechanical stability of the films compared to lignin alone, while also increasing the swelling of the coatings. Similar blends were studied by Mulder *et al.*, who found that glycerol or polyols such as PEG 400 could improve the film forming properties.^120^ The water resistance, on the other hand, was improved by using high molecular weight PEG or crosslinking agents such as Acronal or Styronal (commercial name). On the downside, the biodegradability will be negatively affected by such cross-linking agents, especially acrylates or styrene-based chemistries.

Chemical modification of lignin for coating of superphosphate fertilizers was also conducted by Rotondo *et al.*,^119^ where the technical lignin was either hydroxymethylated or acetylated. Apart from utilizing toxic chemicals in the synthesis, these modifications alone do not pose as a detriment to biodegradability. However, the Rotondo *et al.* also synthesized phenol-formaldehyde resin to coat the fertilizer cores, which could be troubling, as the authors basically suggested adding plastics to the soil. Zhang *et al.* furthermore modified lignin by grafting quaternary ammonium groups onto it.^82^ While the quaternary ammonium may conveniently bind anions and add nitrogen to the soil, some of its degradation products are highly toxic and hence concerning, unless the goal is to add biocides to the soil. A similar approach was done by Li *et al.*,^14^ who synthesized multifunctional fertilizers. First, alkali lignin and NH_4_ZnPO_4_ were mixed and dissolved to produce fertilizer cores, which were further coated with cellulose acetate butyrate and liquid paraffin. A second coating was then applied as a superabsorbent, which was based on alkali lignin grafted with poly(acrylic acid) in a blend with attapulgite. Both the paraffin and poly(acrylic acid) graft should have been avoided due to environmental incompatibilities.

At last, a different application was explored by Nguyen *et al.*, *i.e.*, the encapsulation of photo-liable compounds with a lignin coating layer.^122^ In particular, the authors emulsified the insecticide deltamethrin in a corn oil nanoemulsion with polysorbate 80 and soybean lectin as emulsifier. The droplets were further coated with chitosan and lignosulfonate. The lignin contributed hereby to both the UV-protection of the emulsified insecticide, as well as to its controlled release. This approach is positive in several regards, as only biobased agents were used in the formulation, the lignosulfonates were not chemically modified, and the application drew on some of lignin's inherent properties.

#### 3.3.2. Biomaterials and biomedical applications

A biomaterial, *i.e.*, a material intended for use in or on the human body, must comply with certain requirements. This implies that the material should be biocompatible and should not cause an unacceptable effect on the human body.^123^ However, the definition of biocompatibility has been debated in the literature. In addition to a modified definition of “biocompatibility”, Ratner proposed “biotolerability” to describe biomaterials in medicine.^124^ Biocompatibility was defined by “the ability of a material to locally trigger and guide nonfibrotic wound healing, reconstruction and tissue integration”, while biotolerability was proposed to be “the ability of a material to reside in the body for long periods of time with only low degrees of inflammatory reactions”. Novel biomaterials developed for biomedical applications could be defined by these terms with the target of limited fibrotic reactions,^125^ and lignin may be within this group of biomaterials. Lignin is a material derived from biobased resources, with attractive properties for biomedical use, primarily with antioxidant and antibacterial characteristics. The antioxidant property of lignin is dependent on the phenolic hydroxy groups capable of free-radical scavenging. The antimicrobial effect is also caused by the phenolic compounds.^126^ As expected, the antibacterial, antioxidant and cytotoxic properties may also depend on the type of lignin.^127,128^ For example, kraft lignin has been found to have less antibacterial properties compared to organosolv lignin due to the larger methoxyl content in organosolv lignins.^127^

**Fig. 12:**
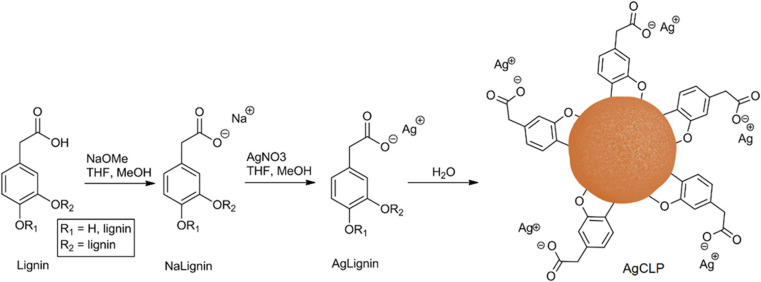
Simplified schematic of the production of silver-doped lignin nanoparticles.^129^

Several authors have attempted to draw on lignin's antibacterial and antiviral properties, which could be useful in surfaces for biomaterials and biomedical applications. Antimicrobial coatings were, for example, prepared by Lintinen *et al. via* deprotonation and ion exchange with silver,^129^ as shown in Fig. 12. Jankovic *et al.* also developed such surfaces by flash-freezing a dispersion of organosolv lignin and hydroxyapatite with or without incorporated silver.^130^ After freezing, the samples were dried by cryogenic multipulse laser irradiation, producing a non-cytotoxic composite, which were further tested on their inhibitory activity. A similar approach was taken by Eraković *et al.*^131^ The authors prepared silver doped hydroxyapatite powder, which was then suspended in ethanol with organosolv lignin and coated *via* electrophoretic deposition onto titanium.^131^ This composite showed sufficient release of silver to impose antimicrobial effect, while posing non-toxic for healthy immunocompetent peripheral blood mononuclear cells at the applied concentrations. However, the use of silver has caused some environmental concerns that should be addressed.^132^ As an alternative, copper has been reported with better antibacterial effect than silver, which to our knowledge is currently unexplored in antimicrobial lignin complexes.^133^

Lignin-titanium dioxide nanocomposites were prepared by precipitation from solution and tested for their antimicrobial and UV-blocking properties.^134^ The authors concluded that the lignin could function as the sole capping and stabilization agent for the titanium dioxide nanocomposites. Better performance of the nanocomposites for antioxidant, UV-shielding, and antimicrobial properties was reported, as compared to the lignin or titanium dioxide alone.

Kraft lignin and oxidized Kraft lignin were processed into colloidal lignin particles and coated with β-casein, which was further cross-linked.^29^ This work aimed to produce biomaterials and bio-adhesives, where the colloidal lignin acted as the scaffold intended for the synthesis of bio-compatible particles. However, no assessment of biocompatibility of the generated complexes was performed. Hence, the question remains whether this approach may be suitable for biomedical applications.

According to Dominguez-Robles, there are various additional biomedical applications, in which lignin could be promising, *e.g.*, as hydrogels, nanoparticles and nanotubes, for wound healing and tissue engineering.^135^ The interest in lignin for biomedical applications was also emphasized by the increasing amount of publications related to lignin applied as a functional material for tissue engineering, drug delivery and pharmaceutical use.^135,136^ However, the reported studies on lignin for biomedical applications is still limited and various challenges will need to be overcome to advance in this area. These are especially regarding relevant assessment of lignins toxicological profile and biocompatibility. Not to mention the large variability of lignins when it comes to the source of lignin, the fractionation processes and posterior modification, which may affect the chemical structure, homogeneity, and purity.

#### 3.3.3. Wastewater treatment

Different researchers have formulated lignin-based materials, which were designed for the purification of dye containing wastewater.^16,102,137^ Suitability for such applications is principally derived from the chemical similarities, which exist between lignin and many dyes, *i.e.*, an abundance of heteroatoms and aromatic moieties. Such adsorbents can be produced *via* deposition on silica gel,^102^ coating onto carbon particles,^16^ carbonization,^43^ sorption and co-precipitation.^138^ Development of such materials is generally positive, as it draws on the unique composition of lignin.

A different technological approach within the same area would be membranes. Layer-by-layer assembly is a frequently used technique, in which polyanionic lignosulfonates or sulfonated Kraft lignin are combined with a polycation. Multiple bilayers are made by stepwise application of the polyelectrolytes, *e.g.*, by immersion in a solution of polyanion first, followed by drying and immersion in a solution of polycation. This approach was used by Shamaei *et al.* as an antifouling coating for membranes, improving the treatment of oily wastewater.^139^ Gu *et al.* used lignosulfonates and polyethyleneimine on a polysulfone membrane, which successfully repelled the adsorption of proteins.^15^ While the preparation is straight-forward, the long-term stability of such coatings also must be demonstrated. Both the lignin and (poly-)cation were water-soluble, so the coating would be pointless if washed away with the retentate.

#### 3.3.4. Packaging applications

Packaging applications can benefit from lignin-containing surfaces in various regards. Improvements in the water-resistance, water and oxygen barrier, and mechanical strength of cellulose-based substrates have been reported.^35,107,108,140^ A more detailed summary of these materials is given in Section 3.3.1. The lignin may also serve as an oxygen scavenger. To implement this, several authors have formulated coatings that include both lignin and laccase enzyme.^113–115^ At last, the antibacterial and UV-shielding properties of lignin have been mentioned as beneficial contributors.^83,107,110^ While the studies demonstrate feasibility on a technological level, there are other factors that must be considered as well. Long-term stability and migration of the coatings is rarely addressed, despite this being a crucial parameter in food packaging. In other words, one must be sure that no detrimental substances are transferred to the food. Some foods release both water and fat, for which lignin is in theory a good match, as it is soluble in neither. Packaging of non-foods generally poses less harsh requirements. The requirements on price per volume are greater; however, the pricing of technical lignin should be competitive.

#### 3.3.5. UV-protection

The polyaromatic backbone of lignin provides extended absorption at sub-visible wavelengths of light. UV-shielding applications hence draw on one of the intrinsic properties of lignin. One example would be the development of natural sunscreens *via* hydroxylation of titanium oxide particles, during which lignosulfonates were added.^141^ Unsurprisingly, it was concluded that the lignin enhanced the UV-blocking effect of the titanium oxide particles. Another publication explored sunscreens, where nanoparticle-size lignin was added to commercial lotion formulations.^142^ The UV absorbance was improved by both nanoparticle formation and pretreatment with the CatLignin process. The latter was explained by partial demethylation and boosting of chromophoric moieties. While lignin may conveniently replace fossil-based and non-biodegradable UV actives, other factors also need to be tested for such a product to become feasible, *e.g.*, non-hazardousness, safety for human contact, and skin tolerance.

Other applications that can profit from this property include UV-protective clothing,^91^ packaging materials,^83,107^ agrochemical formulations,^122^ and personal protective equipment.^134^ It should be mentioned, however, that enhanced UV absorbance is not always beneficial, as it can also lead to faster degradation of the lignin-containing materials.^12^

### 3.4. Lignin as part of thermoplastic materials

Lignin is a thermoplastic material with glass-transition temperatures in the range of 110–190 °C.^24^ As such, it is straight forward to use technical lignin as a filler material in, *e.g.*, thermoplastics or bitumen admixtures.^38^ Potential advantages of lignin in thermoplastic polymer coatings have been discussed by Parit and Jiang,^21^*i.e.*, by adding UV-blocking and antioxidant activity as required in packaging applications. In general, the addition of lignin in thermoplastics can increase stiffness, but at the expense of extensibility.^38^ Chemical modification (alkylation) may be required to improve both tensile stiffness and strength of olefinic polymers.^26^ On the other hand, lignin's amphiphilic make-up can impart advantages, *e.g.*, by improving the adhesion of polypropylene coatings.^143^ Another example would be the use of lignin in biocomposites from polypropylene and coir fibers.^45^ While no significant effect on tensile strength of the composites was found, adding lignin reportedly delayed the thermal decomposition.

Coatings with polymers are frequently used to protect the mechanical integrity of the underlying substrate. For added lignin to be advantageous, the mechanical characteristics of the polymer blend must hence be improved. While publications in this area frequently focus on the added functionalities, some also reported improvements in the mechanical strength of the coatings.^12,26^ On the downside, the addition of lignin is often limited to low ratios and chemical modification may be required.^12^ These factors can limit the overall sustainability gain, which biopolymers have over fossil-based polymers and fillers.

**Fig. 13:**
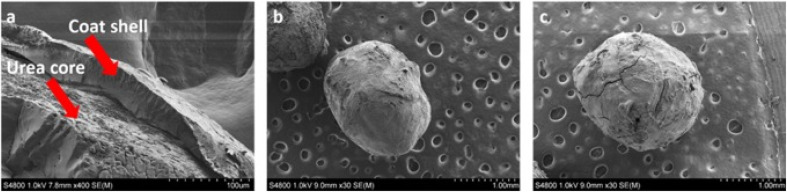
SEM images of PLA-lignin coated urea pellets showing (a) the coating layer over urea core and the coated pellet (b) before and (c) after urea release to surrounding water. This figure has been adapted/reproduced from ref. 144 with permission from Elsevier B.V., copyright 2017.

At last, slow-release fertilizers can be prepared from thermoplastics and lignin. Li *et al.* blended poly(lactic acid) (PLA) with Kraft lignin samples, some of which had been chemically modified by esterification or Mannich reaction.^144^ Urea particles were then coated by solvent casting or dip-coating, where the alkylated lignin yielded improved barrier properties and better compatibility with PLA. Microscope images of the coated urea particles are shown in Fig. 13. While lignin-PLA blends can potentially be more biodegradable than lignin-based resins, the biodegradation in soil may still be insufficient. Our recommendation is hence to favor blends of unmodified lignin with biopolymers, such as starch, cellulose, or carrageenan, as this will not contribute to microplastics pollution.

### 3.5. Lignin as a precursor to thermosets

The four most common applications of lignin in thermosets are polyurethane, epoxide resins, phenolic resins, and polyesters.^11^ Unsurprisingly, formulations of lignin-based thermoset coatings are often derived from such chemistries. The lignin can also be rendered compatible with other formulations, *e.g.*, with polyacrylates by grafting with methacrylic acid.^145^ Such grafting reactions are indeed instrumental to overcome some of the traditional challenges of lignin;^146^ however, they can also be accompanied by unwanted side-effects, such as poor biodegradability.

#### 3.5.1. Lignin-based polyurethane coatings

Lignin utilization in polyurethanes is done as polyol replacement, where lignin's hydroxyl groups are reacted with isocyanate groups acting as cross-linker. The lignin may even be soluble in the polyol, which aids straight-forward substitution. Lignin derivatization to improve the compatibility and performance includes hydroxyalkylation (*e.g.*, with propylene oxide, propylene carbonate, or epichlorohydrin), esterification with unsaturated fatty acids, methylolation, and demethylation.^28^

Chen *et al.* blended alkali lignin and PEG, which were further polymerized with hexamethylene diisocyanate in presence of silica as leveling agent.^147^ Experiments were limited to 60 wt% lignin, as higher ratios yielded an embrittlement. The mixtures were processed into films, which showed some potential for biodegradation. These results indeed corroborated by other authors, which also state that lignin incorporation in polyurethanes yielded a limited degree of biodegradability.^148^ A different approach was made by Rahman *et al.*,^149^ who synthesized waterborne polyurethane adhesives with aminated lignin. The tensile strength and Young's modulus improved with increasing ratios of aminated lignin, which could be due to an increased cross-linking density. Still, the overall percentages of lignin in the coatings were comparably low, as the authors added only between 0–6.5 mol% lignin. It is curious to note that the authors proclaimed better storage stability of aminated lignin dispersions, yet only the weathering resistance of the final coating was measured.

Some of the challenges with lignin in polyurethane materials include reactivity and a high cross-linking density. Due to the latter, polyurethane formulations are frequently limited to low percentages of lignin, typically 20–30 wt% at max, as higher ratios can yield brittle and low-strength materials.^150^ One approach is to increase the degree of substitution is depolymerization of lignin, but other chemical modifications or fractionations may be equally applicable. In this context, the work by Klein *et al.* should be mentioned, who reported polyurethane coatings with lignin ratios of up to 80%.^151^ A comparably low curing temperature of 35 °C was used, which could also entail incomplete reaction. Curiously, there is no data on the mechanical strength of the films. In addition, the authors measurements of hydroxyl groups *via* ISO 14900 and ^31^p-NMR are widely divergent. In two other publications by the same author, the antioxidant properties and antimicrobial effect of such films were studied.^13,152^ In a different study, methyl-tetrahydrofuran was used to extract the low-molecular weight portion from Kraft lignin.^153^ The authors used between 70 to 90 wt% lignin in the final formulation at NCO/OH molar ratios of 0.16–0.04. While providing a good adhesive strength, the films elastic modulus is within the same range of the fractionated lignin, whereas no information on the material strength was provided. It would thus appear that the elevated cross-linking density may be circumvented, *i.e.*, simply by reacting only a sub-fraction of the available hydroxyl groups of lignin. Still, it has yet to be demonstrated that such coatings are also competitive in mechanical strength and abrasion resistance.

#### 3.5.2. Lignin-based phenolic resin coatings

Lignin may also be used as a phenol-substituent in phenol-formaldehyde resins.^20^ This approach was utilized by Park *et al.* to produce cardboard composites by spray coating.^154^ The authors reported that lignin purification by solvent extraction yielded better results than by acid precipitation. Substituting with 20–40 wt% lignin surprisingly accelerated the curing kinetics, compared to the lignin-free case. The coated cardboard showed lower water absorption; however, the contact angle was also lower, which could be due to a change in surface chemistry and morphology. It would be interesting to study even higher degrees of substitution and to delineate with the mechanical strength. Still, it appears that coatings with lignin-phenol-formaldehyde have so far been aimed at providing a water-barrier. For example, the work by Rotondo *et al.* coated superphosphate fertilizers with hydroxymethylated lignin resins,^119^ which significantly slowed the phosphate release.

#### 3.5.3. Lignin-based epoxy resin coatings

Similar to lignin-containing polyurethanes, epoxy resins also target a reaction with the hydroxyl groups. In analogy to that, chemical conditioning such as depolymerization can potentially improve the final material. For example, Ferdosian *et al.* tested different ratios of depolymerized Kraft or organosolv lignin in conventional epoxy resin formulations.^155^ The authors showed that large amounts of lignin retarded the curing process particularly in the late stage of curing. At the right dosage (25%), the lignin-based epoxy exhibited better mechanical properties than the neat formulation, while improving adhesion on stainless steel. Both effects appear plausible considering lignins macromolecular and polydisperse composition. In this context, a recent patent by Akzo Nobel should also be mentioned, which describes the use of lignin and potential epoxy crosslinker for functional coatings.^156^ A different approach was chosen by Hao *et al.*, who carboxylated Kraft lignin first, followed by its reaction with PEG-epoxy.^157^ The coatings possessed a lignin content of 47%. In addition, the self-healing ability was demonstrated by transesterification reaction in presence of zinc acetylacetonate catalyst.

Crosslinking of nanoparticles is an interesting approach, as the coagulation to nanoparticles may favor a different ratio of functional groups at the surface than in the bulk lignin. In addition, this approach can produce composite materials, which exhibit different characteristics than a homogeneous polymer. For instance, Henn *et al.* combined lignin-nanoparticles with an epoxy resin, *i.e.*, glycerol diglycidyl ether, to treat wood surfaces.^106^ The coatings showed nano-structured morphology, which still preserved the breathability of the wood, hence drawing advantage from lignin's nanoparticle formation. Zou *et al.* coprecipitated softwood Kraft lignin together with bisphenol-a-diglycidyl ether to produce hybrid nanoparticles.^158^ The particles were either cured in dispersion for further cationization or directly tested in their function as wood adhesives. The use of lignin-based nanoparticles in curable epoxy resins is hence promising, as it can generate new functionalities, but the maturity of this technology still needs to be advanced.

#### 3.5.4. Lignin-based polyester coatings

**Fig. 14:**
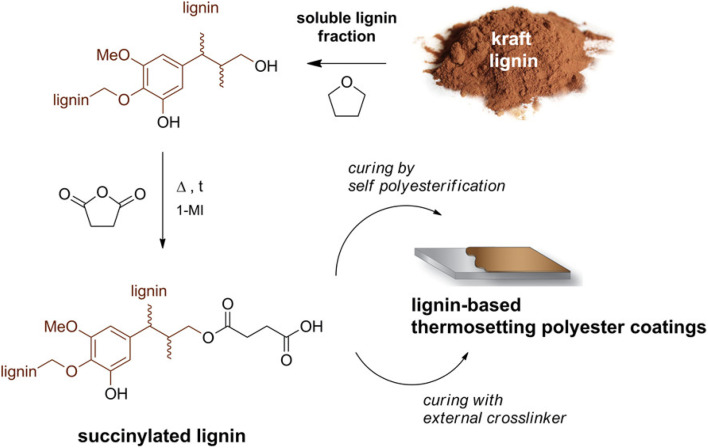
Schematic of producing lignin-based thermosetting polyester coatings. This figure has been adapted/reproduced from ref. 19 with permission from American Chemical Society, copyright 2018.

While the use of lignin in polyester coatings is technologically feasible, few publications were found to this topic. One reason for this could be the slow reaction kinetics of direct esterification. Coupled with lignin's structure and chemistry, polyester-based coatings would be less straight forward than polyurethanes or epoxy resins, which involve highly reactive coupling agents. As discussed previously, chemical modification of lignin may improve this circumstance, for example by depolymerization or introduction of new reactive sites. As such, oxidative depolymerization and subsequent membrane fractionation has been suggested to produce a raw material, which can be utilized in subsequent polyester coatings.^159^ A second example would be solvent-fractionated lignin, which has been carboxylated by esterification with succinic anhydride.^19^ As illustrated in Fig. 14, the modified lignin reportedly underwent self-polymerization, where the grafted carboxyl groups reacted with residual hydroxyl groups on the lignin. Development in this area has potential, as polyesters tend to exhibit better biodegradability than polyolefins.

#### 3.5.5. Lignin-based acrylate coatings

Lignin-based acrylates rely on the grafting of acrylate moieties, as these are not inherent to lignin. For example, methacrylation of Kraft lignin was done to produce UV-curable coatings.^145^ The authors concluded that incorporating lignin into the formulation improved thermal stability, cure percentage, and adhesive performance. An elaborate study on aging of lignin-containing polymer materials was conducted by Goliszek *et al.*^30^ The authors grafted Kraft lignin with methacrylic anhydride and further polymerized the product with styrene or methyl methacrylate. Low amounts of lignin (1–5%) showed incorporation into the network, whereas higher concentrations showed a plasticizing and more heterogeneous effect. High lignin loadings also enhanced the detrimental effects of aging, which may seem counterintuitive, as other reports frequently state a UV-protective ability of lignin. Still, increase absorption of UV light can also amplify the detrimental effects thereof. A combination of epoxy and acrylate was used to develop dual-cured coatings with organosolv lignin.^160^ The lignin was first reacted with epoxy resin and subsequently with acrylate to form a prepolymer. In a second step, the prepolymer was mixed with initiators and diluent to be coated onto tinplate substrates. All in all, lignin-based acrylate coatings appear to have reached sufficient technological maturity, yet the advantage of adding lignin is sometimes unclear.

#### 3.5.6. Other approaches

Szabo *et al.* grafted Kraft lignin with *p*-toluenesulfonyl chloride, whose product was then grafted onto carbon fibers.^161^ The results suggested an improved shear tolerance of the modified carbon fibers in epoxy or cellulose-based composites. Silylation was furthermore performed of Kraft lignin, which was further co-polymerized with polyacrylonitrile.^162^ The authors concluded that silylation improved the compatibility for surface coatings and films.

## 4. Lignin in technical applications – a critical commentary

The development high-value products from lignin has been a topic of great interest for some years, and is still gaining popularity.^163^ Added-value applications are being pursued, ranging from asphalt emulsifiers or rubber reinforcing agents, to the production of aromatic compounds *via* thermochemical conversion.^164^ The question arises, however, if including lignin in a coating can really lead to a better overall product? Comparison with state-of-the-art formulations is frequently omitted, benchmarking lignin-based solutions only to a reference case with low performance. “Attributing value to waste” is one of the primary motivations behind lignin-oriented research. For example, bioethanol production from lignocellulose biomass often gives rise to a lignin-rich byproduct. The overall economics of such biorefineries could be improved if the lignin-rich residue could be marketed at a value. Still, to establish a new product on the market, this product also needs to compete with existing solutions in terms of performance and price. This point is often overlooked in literature, in particular concerning lignin-based surfaces and coatings.

Harnessing lignin's inherent properties is key, as this can create synergies and yield an advantage over other biopolymers. It comes to no surprise that the dominant use of technical lignin is in water-soluble surfactants, as polydispersity is a key feature here.^3^ As has been pointed out, the performance of surfactant-blends often outperforms single surfactants in real-world applications, since the mixture can preserve its function over a wider range of environmental conditions. A second example of key properties would be lignin's polyphenolic structure, which is not found in common polysaccharides. Lignin has hence been investigated as a UV blocking additive in *e.g.*, sunscreen products or packaging.^165^ However, compatibility of the resulting product with human- or food-contact is addressed insufficiently by many authors. A similar situation was given in case of lignin as antioxidant additive in cosmetics,^166^ where the dark color and smell may limit the final use.

Compared to cellulose or hemicellulose, lignin has a higher carbon-to-oxygen ratio. Due to this and its polyaromatic structure, it would indeed be a better raw material for producing carbonaceous materials. Research on activated carbon, graphitic carbon, and carbon fibers has indeed being conducted.^167^ A key step toward lignin-based carbon fiber production was identified as removal of β-O-aryl ether bonds.^60^ In addition, the charring ability of lignin has been proposed as a benefit in fire retardants.^168^ Still, lignin-based fire retardants often use chemical modifications, such as phosphorylation. If chemical modification is necessary, the question arises if such chemistries really need to be based on lignin, since other biomacromolecules may possess a higher reactivity and number of reactive sites.

Lignin can be readily precipitated from solution into nanoparticles and nanospheres. Various applications have been suggested based on this, such as functional colloids and composite materials with uses in flame retardancy, food packaging, agriculture, energy storage, and the biomedical field.^169^ A more specific example would be nanoparticulated lignin in poly(vinyl alcohol) films with increased UV absorption.^170^ While this technology appears straight-forward, its final use has yet to be proven.

At last, technical lignin is usually thermoplastic, exhibiting glass-transition temperatures in the range of 110–190 °C.^24^ The use of lignin as polymeric filler or in thermoplastic blends is hence promising. In some cases, chemical modification may be necessary to improve compatibility, *e.g.*, with polyolefins;^26^ however, the use as simple filler material would not necessitate modification. Additional strength could also be derived from added cellulose fibers, which could potentially benefit from added lignin as compatibilizer.

In summary, one needs to build on the inherent properties of lignin, such as polydispersity, poly-aromaticity, a higher C/O ratio than for polysaccharides, and thermoplasticity. Only by utilizing characteristics that set lignin apart from other biopolymers, can solutions be developed that are innovative and market competitive. Chemical modification is a useful tool for tailoring; however, each processing step will add an economical and environmental cost to the final product. In other words, the simplest approach is often the best – something that is frequently disregarded when developing complex synthesis protocols for lignin.

## 5. Summary and conclusion

Functional surfaces and coatings can be formulated in a variety of ways, which includes the use of neat, chemically modified, blended, and cross-linked lignin. This review provides a summary of the current developments in research, where focus was placed on the formulation and final applications.

Overall, coatings with neat lignin or blends of lignin with other active ingredients appear the most practical. Reduced wetting is hereby achieved, as the lignin can alter the surface morphology, hinder mass-transport, and confine swelling of enclosed fibers. The lignin itself is not considered a hydrophobic material, because the contact angle is usually below 90°. On the other hand, hydrophobicity can be induced by plasma surface treatment, blending with other agents, or chemical modification. For the latter, grafting or esterification of lignin with alkyl-containing moieties is a frequently taken approach. Chemical modification may also be used to improve the compatibility with olefinic thermoplastics. Addition of lignin can fine-tune the characteristics thermoplastics and improve adhesion to other materials. On the downside, embrittlement frequently limits this technology to low percentages of lignin. Thermoset coatings with lignin can be based on chemistries such as polyurethanes, phenolic resins, epoxy resins, polyesters, and polyacrylates. Various synthesis routes have been proposed in literature, which can benefit to some degree of the inherent properties of lignin.

Both the formulation and processing depend on the final application of the coating or surface functionalization. The use of lignin with cellulose-based substrates is frequently suggested, as this can yield all-biobased materials. Lignin can improve the resistance to wetting of paper and pulp products. In addition, it can add UV protection and oxygen-scavenging capabilities in packaging applications. Lignin-based surfaces have also been proposed for adsorbents for wastewater treatment, wood veneers, and corrosion inhibitors for steel. The biomedical field has also explored lignin-based biomaterials, which draw on its potential antimicrobial properties. A great number of publications also reports on agricultural uses, where a lignin-based coating may account for slower release of fertilizer. At last, general-purpose polymer coatings can be tailored *via* the inclusion of lignin, and the resistance to fouling of membranes can be improved. All mentioned applications were discussed critically in this review, placing emphasis on the benefit that adding lignin may provide. While introduction of functionalities may be possible, publications frequently do not compare to a well-performing reference case, hence limiting the assessment of the true potential. In addition, the ratio of lignin in thermoset coatings is usually quite low. Higher levels may be achieved after chemical modification, but such synthesis can also have negative implications on the economic and environmental cost of the final product.

In conclusion, the advancement of functional surfaces and coatings with lignin has yielded promising results. However, there also must be a benefit of using lignin compared to other biopolymers or existing petrochemical solutions. Only by harnessing lignin's inherent properties, can solutions be developed that are competitive and value-creating. These properties include lignin's polyphenolic structure, a higher C/O ratio than, *e.g.*, polysaccharide biopolymers, its ability to self-associate into nano-aggregates, and its thermoplasticity. These properties are utilized in some of the reviewed literature, hence providing the ground for new and promising technology in the future.

## Author contributions

Jost Ruwoldt: conceptualization, writing original draft, review, editing & visualization. Fredrik Heen Blindheim: writing & visualization. Gary Chinga-Carrasco: writing, review, editing, visualization, supervision.

## Conflicts of interest

The authors declare no conflict of interest.

## Supplementary Material
